# 3D - Printed Patient Specific Instrumentation in Corrective Osteotomy of the Femur and Pelvis: A Review of the Literature

**DOI:** 10.1186/s41205-020-00087-0

**Published:** 2020-11-10

**Authors:** Njalalle Baraza, Chris Chapman, Sima Zakani, Kishore Mulpuri

**Affiliations:** 1 Department of Orthopaedic Surgery, BC Children’s Hospital, 1D.66-4480 Oak Street, Vancouver, BC V6H 3V4 Canada; 2 Department of Surgery, Aga Khan University Hospital, Nairobi, Kenya; 3 Department of Orthopaedic Surgery, Brookdale Hospital Medical Center, Brooklyn, NY USA; 4 BC Children’s Hospital, Vancouver, BC Canada; 5 Department of Orthopaedics, University of British Columbia, Vancouver, BC Canada

**Keywords:** Patient specific instrumentation, 3D printing, Orthopaedic surgery, Femoral osteotomy, Pelvic osteotomy

## Abstract

**Background:**

The paediatric patient population has considerable variation in anatomy. The use of Computed Tomography (CT)-based digital models to design three-dimensionally printed patient specific instrumentation (PSI) has recently been applied for correction of deformity in orthopedic surgery. This review sought to determine the existing application of this technology currently in use within paediatric orthopaedics, and assess the potential benefits that this may provide to patients and surgeons.

**Methods:**

A review was performed of MEDLINE, EMBASE, and CENTRAL for published literature, as well as Web of Science and clinicaltrials.gov for grey literature. The search strategy revolved around the research question: “What is the clinical impact of using 3D printed PSI for proximal femoral or pelvic osteotomy in paediatric orthopaedics?” Two reviewers, using predetermined inclusion criteria, independently performed title and abstract review in order to select articles for full text review. Data extracted included effect on operating time and intraoperative image use, as well as osteotomy and screw positioning accuracy. Data were combined in a narrative synthesis; meta-analysis was not performed given the diversity of study designs and interventions.

**Results:**

In total, ten studies were included: six case control studies, three case series and a case report. Five studies directly compared operating time using PSI to conventional techniques, with two showing a significant decrease in the number of intraoperative images and operative time. Eight studies reported improved accuracy in executing the surgical plan compared to conventional methods.

**Conclusion:**

Compared to conventional methods of performing femoral or pelvic osteotomy, use of PSI has led to improved accuracy and precision, decreased procedure times, and decreased intra-operative imaging requirements. Additionally, the technology has become more cost effective and accessible since its initial inception and use.

**Supplementary Information:**

The online version contains supplementary material available at 10.1186/s41205-020-00087-0.

## Background

Patient specific instrumentation (PSI) has been utilized for nearly two decades since its development by Radermacher et al. [1] Initially, the technology was proposed for application in spinal and pelvic surgery as it provided a patient specific cortical read for instrument positioning and placement without the need for repeated radiographs [1]. The use of these PSI was found to decrease both operating time and the need for intraoperative fluoroscopy.

In our unit, the creation of PSI involves a three-dimensional reconstruction of the relevant anatomical area developed from a preoperative CT scan. The information is then fed into specialized software (MIMICS, Materialise). The required structures (in our case, bone) are then highlighted through a combination of manual and automatic processes, before it is converted into an electronic three-dimensional (3D) model. Next, the procedure is planned, including osteotomy locations, desired correction, and screw and/or pin size and placement. This preoperative plan is then used to create a unique navigation template. The template is then exported in stereolithography (STL) format and sent to the 3D printer, where it is is manufactured using materials that can be appropriately sterilized for use in the operating room. The printed template can subsequently be used to complete the surgical procedure as planned without requirement for any additional tools or equipment such as computer guided navigation.

We set out to determine the extent and utility of PSI’s application within paediatric orthopedic surgery. Our specific area of interest was the effect of PSI use on execution of plan, operative time, fluoroscopy requirement, and overall outcomes of femoral/pelvic osteotomy for congenital or acquired deformity. Our question was “What is the effect of 3D printed individualized template use for proximal femoral or pelvic osteotomy in paediatric patients?”

## Methods

**Fig. 1:**
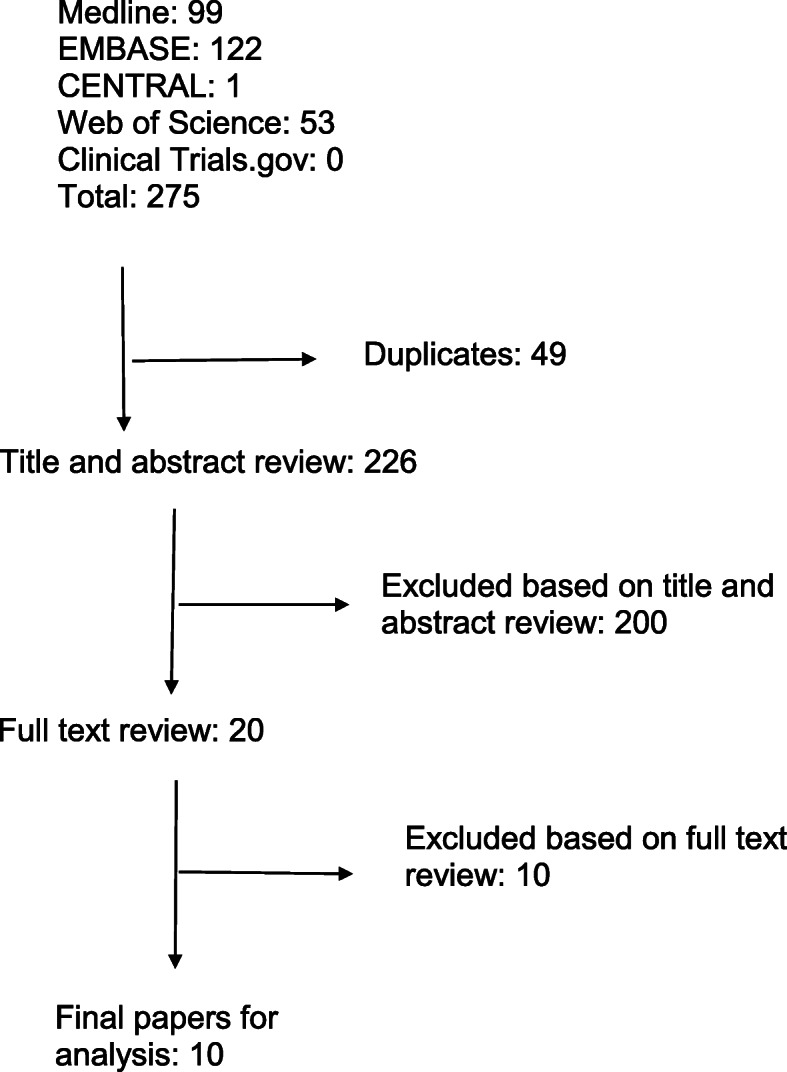
Study Selection from Search Performed 16th Sept 2020

A primary literature search was conducted by our medical librarian with the aim of identifying any studies performed within the last 15 years that examined the use and effect of PSI on deformity correction procedures of the proximal femur or pelvis. This search was limited to paediatric patients and had no language restriction given the limited number of articles available. Our search was conducted using Ovid Medline, Ovid Embase, and CENTRAL, as well as WebOfScience and clinicaltrials.gov for unpublished/grey literature. An example of our search string used in Ovid Medline (reference) can be found in Additional file 1: Appendix 1. Our predetermined inclusion criteria were patients with pelvic or proximal femoral deformity/lesion, use of PSI, femoral/pelvic application of PSI, within 15 years. Exclusion was based on application of PSI to arthroplasty of the pelvis or femur, and their use in adult patients. Our search of the above databases produced 1143 studies. After removal of duplicates and non-paediatric based papers, 226 papers remained. Two independent reviewers, using the predetermined selection criteria, performed title/abstract review and found 20 studies appropriate for full text review. The same reviewers independently performed full-text review and found 10 studies for final inclusion and analysis. Any differences were resolved by consensus after discussion. Figure 1 outlines our study selection. Each selected study was graded for level of evidence using the Oxford Center for Evidence Based Medicine Levels of Evidence Guidelines [2]. Data extraction was performed manually, and included effects on operating time and intraoperative image use, as well as osteotomy, screw positioning, and general clinical outcome. Data were combined in a narrative synthesis; a meta-analysis was not performed given the diversity of study designs and interventions examined. Characteristics of included studies can be found in Table 1.

**Table 1:** Characteristics of Included Studies

Study	Level of Evidence [2]	Sample Size	Setting	Relevant Reported Result Domains	*p* value
Zheng et al., Sci Rep, 2017 [3]	3	12 cases v 13 controls	Proximal Femoral Varus and Shortening Osteotomy following DDH	Reduced OR Time, intraoperative Imaging and better correction achieved relative to plan	*p* < 0.05
Zheng et al., Int J Comput Assist Radiol Surg, 2017 [4]	3	11 patients	Accuracy of placement of screws in paediatric hip plate	OR Time Intraoperative Imaging Template Fit/Match to Cortex Screw Placement and Trajectory	no comparison group
Zhou et all., Medicine 2016 [5]	4	5 Cadaveric Specimens, 10 Hemi-Pelvises	Bernese Peri-Acetabular Osteotomy	Computer simulated osteotomy vs 3D printed patient specific guide in periacetabular osteotomy	*p* > 0.05
Sallent et al., Bone Joint Res, 2017 [6]	3	5 Cadaveric Specimens, 10 Hemi-Pelvises	Pelvic Bone Tumour Resection	Deviation of Osteotomy from Plan	*p* < 0.032
Tong K et al., Journal of Southern Medical Uni, 2013 [7]	4	13 hips	Steele Osteotomy for DDH	Accuracy of osteotomy using Reverse Engineered templates	No statistical analysis
Shi, Sun [8]	3	14 controls v 15 cases	Femoral corrective osteotomy in DDH	Accuracy of correction, intraopetrative time, number of fluoroscopic images	*p* = 0.0003
Cherkasskiy et al. [9]	3	15 patients	Proximal femoral osteotomy following SCFE - comparison using 3D model, no model and senior surgeons (5 pts. per group)	Operative time, fluoroscopy time	*p* = 0.40, 0.30
Bortulev et al. [10]	3	15 cases v 15 controls	Triple pelvic osteotomy	Accuracy of correction	*p* < 0.05
Jentzsch et al. [11]	4	4	Pelvic tumour resection	Tumour free resection margins,	no statistical analysis
Buhler T et al. [12]	5	1	Use of 3D printed template guide for osteotomy of the femur to correct malunion	Accuracy of correction	no statistical analysis

## Results

Ten articles were considered eligible for final inclusion. Six were case control studies [3, 5, 6, 8–10], three were case series [4, 7, 11], and one was a case report [12]. Of the ten articles in the final inclusion, three reported directly on operative time and intraoperative imaging use. All three found a decrease in the number of intraoperative images required for completion of the planned procedure using PSI compared to conventional techniques [3, 8, 9]. Additionally, all three found a decrease in operative time compared to conventional techniques when PSI were used, although in one of the studies this difference was not found to be statistically significant [9]. In one of the studies the clinical outcome was better in the group that utilised PSI, but again this was not statistically significant [3]. Simple visual examination of the data in Table 2 demonstrates that both the number of intraoperative images required and operative time were markedly reduced compared to conventional surgery for the same procedures.

**Table 2:** Operative Time and Intraoperative Imaging for PSI vs Conventional Techniques

Study	LoE [2]	Sample Size	Setting	OR Time (min)		Intraoperative Imaging	
				Conventional	PSI	Conventional	PSI
Zheng et al., Sci Rep, 2017 [3]	3	12 cases v 13 controls	Proximal Femoral Varus and Shortening Osteotomy following DDH	46.92 ± 11.51	21.08 ± 4.64	6.69 ± 1.49	3.92 ± 0.90
Zheng et al., Int J Comput Assist Radiol Surg, 2017 [4]	3	11 patients	Accuracy of placement of screws in paediatric hip plate	57.15 ± 9.25	26.50 ± 4.07	11.85 ± 3.15	6.00 ± 0.73
Shi Q, Sun D. JOSR 2020 [8]	3	15 cases v 14 controls	Femoral corrective osteotomy in DDH	37.3 ± 8.8	20.6 ± 3.9	8.1 ± 2.8	3.0 ± 1.4
Cherkasskiy et al. J Child Orthop, 2017 [9]	3	15 patients	Proximal femoral osteotomy following SCFE	170.4 ± 876.4	125.8 ± 25.4	0.6 ± 0.4	0.3 ± 0.3

*LOE* level of evidence, *OR* operating room, *DDH* developmental dysplasia of the hip, *OA* osteoarthritis

Six studies commented on template fit, placement and accuracy of osteotomy, and all found that once the template was positioned, the match of the cortical topography was good based on the 3D reconstruction [4, 5, 7, 8, 10, 12]. One study reported on screw placement and trajectory, finding that PSI improved accuracy of screw start points and trajectories compared to conventional methods [4]. A single study reported on linear/angular deviation of the osteotomy from plan finding improvement in both when PSI was used compared to conventional methods [6]. Two studies reported on correction achieved relative to planned procedure found greater agreement with the operative plan when PSI was used compared to conventional means [3, 5]. Three case series/reports were included that did not report directly on operative time or imaging [7, 11, 12], however they found that PSI use improved clear margins in pelvic tumor excision [11], led to reliably accurate osteotomies [7], precise correction of deformity and excellent clinical outcomes [12].

## Discussion

The planning of complex paediatric orthopaedic surgery is quite often daunting. In addition to speaking to and preparing both parent and patient for a major operation, there are thoughts of surgical access to the affected area, blood loss and other possible morbidity inducing complications, location of bony cuts if needed, degrees of correction, parameters of acceptability, fixation methods and post-operative rehabilitation and mobilisation. Any technology that can ease the burden on the clinician’s mind whilst ensuring a more optimal outcome for the patient should be explored and if proven to be beneficial, considered as an additional resource in the treatment armamentarium.

Since its inception, 3D-printed PSI has not had widespread application within paediatric orthopedic surgery. This trend seems to be changing though; based on the limited available evidence, PSI appears to be effective for application in deformity correction about the femur and pelvis. From our review, it was found to be simple to use, facilitated clear pre-operative planning and effective intraoperative implementation of that plan (see Additional file 2: image 1) while removing the need for continuous intraoperative fluoroscopic monitoring - this in particular leading to a further reduction in operative times and perhaps more importantly lessening the potential harmful effects of unnecessary radiation exposure to patient and operating team.

According to the Radiological Society of North America (RSNA) 3D printing Special Interest Group (SIG) guidelines for medical 3D printing and appropriateness for clinical scenarios, the use of 3D printed models can positively impact numerous metrics associated with musculoskeletal interventions, including patient and physician satisfaction, operative time, blood loss, and costs associated with patient-centered decision making regarding management of complex disease. In particular, the RSNA identified hip dysplasia, complex acetabular fractures and tumour surgery as areas of practice that would most benefit from the usefulness of this emerging technology [13].

In keeping with this growing awareness, there have been studies detailing the efficiency of 3D anatomic printed models over 3D CT scanning in the operative treatment of paediatric hip disorders. Liu et al. [14] found that in the patients with developmental hip dysplasia (DDH) who had surgery templated using the 3D anatomic printed model, there was a decrease in surgery time, post-operative recovery time, a lower (better) acetabular index and centre edge angle (radiographic parameters of correction). Hedelin et at [15] in his case study showed a strong correlation between the CT scan of the patient’s pelvis and a CT scan of the 3D printed model, showing the reliability of these models in teaching and their usefulness in assisting surgeons with mentally visualising complex procedures, as well as a possible tool for quality control in assessment of postoperative correction.

A further use of 3D printed models was found by Cherkasskiy et al. [9] when he utilised this technology to perform three-plane proximal femoral osteotomy to correct the extension, varus, and external rotation deformity post slipped capital femoral epiphysis, and compared to his colleagues who did not use the models, found that he had reduced operative time and fluoroscopy exposure. Because of the small numbers in the study, the difference in times were not found to be statistically significant but for an emerging technology his study adds to the body of evidence strengthening the case of its clinical relevance.

As the technology and materials used continue to advance, 3D printed models have recently been used for ‘trial runs’ in performing complex paediatric pelvic osteotomies. In a comparative study, Caffrey et al. used 3D printed models to determine the amount of movement of the acetabulum and coverage of the femoral head when performing Pemberton, Dega and San Diego osteotomies, and they did show the utility of 3D printing in paediatric orthopaedics, and its value in determining the most appropriate surgical osteotomy in the treatment of hip dysplasia [16].

With regards to patient specific instrumentation (PSI) derived from 3D models, a study by Lee et al. [17] showed increased accuracy in femoral stem version using CT based navigation with PSI than the conventional technique of visual assessment during hip arthroplasty. In a similar study assessing acetabular components in hip replacement surgery following hip dysplasia, Zhang et al. [18] found more accurate placement of the components when using patient specific instrumentation derived from 3D printed models.

Important though the above articles are in helping gain an appreciation of 3D printing and the wide ramifications of its use, our study question was specifically looking at the application of 3D printed patient specific instrumentation in paediatric orthopaedic surgery. All of the ten articles included in our final review reported advantages of using 3D printed models and derived patient specific templates. It would seem that once knowledge of its usefulness becomes widely accepted, and from the growing evidence this is likely to be in the near future, PSI may become standard practice in complex paediatric orthopaedic surgery.

In a recent review article however, Run-zhi Xia et al. [19] highlighted the drawbacks of using PSI - there is a paucity of high quality evidence detailing this particular use of 3D printing, relatively short follow up, lack of control groups in a number of the studies that do exist, and statistically insignificant improvements in operative times and limb alignment. It is true that 3D printed PSI has poorly defined indications at present, and as was highlighted, there are only a limited number of published studies, each with significant variation on reporting of outcomes.

There is also the risk of overdependence on PSI, and in the critical appraisal of the paper by Zheng et al. [4] there was the revelation that despite PSI use, screw trajectories may still deviate from plan if appropriate care is not taken during performance of the procedure. This may be in part due to difficulty in guide positioning especially on topographically uniform areas such as diaphyseal bone. Additionally, depending on location, soft tissue and risk of injury to certain structures can prohibit required exposure of cortical surfaces necessary for accurate placement.

Another potential stumbling block in the adoption of PSI is cost. There is no doubt that 3D printing technology can be thought of as expensive when one considers the price of printers and the materials needed to make the models and templates. Even though the upfront costs of 3D printing and generation of patient specific instrumentation may seem high, it offsets a number of other expensive surgically related events making it actually a cost saving investment in the long run. Ballard et al. [20] showed that in orthopaedic surgery the savings in operative time from having 3D anatomic models and surgical guides made it a downstream cost effective intervention, and of value to health systems. This is not to mention the costs saved by avoiding potential complications, and generating greater expertise among the clinicians.

In our opinion, the relative absence of high quality evidence should not deter surgeons from what appears to be a useful adjunct to achieving excellence in surgical procedures. The valid criticisms raised by Xia et al. [19] can be put down to the fact that 3D printing is an emerging technology, and as more centres adopt its use, conduct audits and set up databases, the paediatric orthopaedic community can expect more quality trials which will deepen research into helping define and refine its use. All the authors of the referenced studies found that 3D printed PSI is of immense benefit to surgeons. It improved their appreciation of the anatomy, engendered deeper thinking about the cases they were about to perform during preoperative planning, made potentially technically challenging procedures more straightforward by providing a tactile and visual template of the operation for pre-operative preparation which in turn led to an increased chance of a predictably ideal outcome compared to conventional techniques. It is also a great aid to those who do not have the benefit of or access to certain resources provided by tertiary centres e.g. navigation operating.

## Conclusion

From the available evidence, we found that in the context of femoral or pelvic osteotomy in paediatrics, PSI use has demonstrated improved accuracy and precision while decreasing procedure times and intraoperative imaging requirements compared to conventional methods. Additionally, the technology has become less costly and more accessible since its initial inception and use. Given the promise of this relatively new technology in the field of paediatric orthopedics, there is a limited amount of data on its application and effectiveness. Further larger scale studies will need to be completed in order to verify the utility, specific applications and indications of PSI in paediatric patients requiring orthopaedic surgery.

## Supplementary Information

**Additional file 1.** Appendix 1.

**Additional file 2.** Image.

## Data Availability

The datasets used and/or analysed during the current study are available from the corresponding author on request.

## Abbreviations

CT: Computerised tomography
3D: Three dimensional
PSI: Patient specific instrumentation
DDH: Developmental dysplasia of the hip
