# Supplementary material for: 3D - Printed Patient Specific Instrumentation in Corrective Osteotomy of the Femur and Pelvis: A Review of the Literature
Source: 3D Print Med. 2020 Nov 10;6:34. doi: 10.1186/s41205-020-00087-0 (PMC7653713; doi:10.1186/s41205-020-00087-0)
Supplement: Supplementary file 1 — Additional file 1. Appendix 1. [file 41205_2020_87_MOESM1_ESM.docx]

**Search Strategies**

***MEDLINE***

Database: Ovid MEDLINE(R) and Epub Ahead of Print, In-Process & Other Non-Indexed Citations, Daily and Versions(R) <1946 to September 15, 2020>

Search Strategy:

--------------------------------------------------------------------------------

1 Printing, Three-Dimensional/ (5507)

2 Imaging, Three-Dimensional/ (72017)

3 ((3D or 3 D or 3 dimensional or 3dimensional or three D or threeD or three dimensional or threedimensional) adj3 (print* or model? or planning)).tw,kf. (44660)

4 or/1-3 (110989)

5 osteotomy/ (30181)

6 osteotom*.tw,kf. (33933)

7 5 or 6 (44622)

8 Femur/ (41632)

9 (femur? or femoral or trochanter?).tw,kf. (169420)

10 pelvis/ (22833)

11 pelvic bones/ or acetabulum/ or ilium/ or ischium/ or pubic bone/ (29951)

12 (pelvi# or acetabul* or iliac or ilium or ischial or ischium or pubic bone).tw,kf. (196635)

13 hip/ or hip joint/ (37798)

14 Hip Dislocation, Congenital/ (7819)

15 (hip or coxa).tw,kf. (143347)

16 or/8-15 (467132)

17 and/4,7,16 (293)

18 exp Child/ or exp "Congenital, Hereditary, and Neonatal Diseases and Abnormalities"/ or exp infant/ or adolescent/ or exp pediatrics/ or child, abandoned/ or exp child, exceptional/ or child, orphaned/ or child, unwanted/ or minors/ or (pediatric* or paediatric* or child* or newborn* or congenital* or infan* or baby or babies or neonat* or pre-term or preterm* or premature birth* or NICU or preschool* or pre-school* or kindergarten* or kindergarden* or elementary school* or nursery school* or (day care* not adult*) or schoolchild* or toddler* or boy or boys or girl* or middle school* or pubescen* or juvenile* or teen* or youth* or high school* or adolesc* or pre-pubesc* or prepubesc*).mp. or (child* or adolesc* or pediat* or paediat*).jn. (5117021)

19 17 and 18 (99)

20 17 not 19 (194)
