# Supplementary material for: 3D - Printed Patient Specific Instrumentation in Corrective Osteotomy of the Femur and Pelvis: A Review of the Literature
Source: 3D Print Med. 2020 Nov 10;6:34. doi: 10.1186/s41205-020-00087-0 (PMC7653713; doi:10.1186/s41205-020-00087-0)
Supplement: Supplementary file 2 — Additional file 2. Image. [file 41205_2020_87_MOESM2_ESM.zip › PSI image caption.docx]

Example of 3D surgical planning with patient specific instrumentation.

(A) A 3D composition of the femoral head and condyles is performed from CT images of the patient; (B) thus, the angle of femoral anteversion is determined more realistically and accurately; (C,D) the bespoke guide is manufactured using 3D printing technology. It consists of two pieces (distal and proximal) between which there was a space for cutting bone during osteotomy. Both pieces are provided with holes for temporary bone fixation by monocortical screws or wires. The proximal part presented one slot [1] and the distal part had two slots [2,3], in which an auxiliary piece (red) was placed to join both pieces; (C,E) the union of the two parts through the slots number 1 and number 2 adapted the guide to femur at its initial position; (D,F) the guide was designed so that by rotating the distal femoral part and aligning both pieces through slots 1 and 3, a bone external rotation of 40° occurred, necessary for the correct alignment of the femur.

Reproduced with kind permission

Fiz N, Delgado D, Sanchez X, Sanchez P, Bilbao AM, Oraa J, Sanchez M. Application of 3D technology and printing for femoral derotation osteotomy: case and technical report. [Ann Transl Med](https://www.ncbi.nlm.nih.gov/pmc/articles/PMC5673778/). 2017 Oct; 5(20): 400. doi: [10.21037/atm.2017.07.03](https://dx.doi.org/10.21037%2Fatm.2017.07.03) PMCID: PMC5673778 PMID: [29152500](https://www.ncbi.nlm.nih.gov/pubmed/29152500)
