# Supplementary figures and images for: 3D - Printed Patient Specific Instrumentation in Corrective Osteotomy of the Femur and Pelvis: A Review of the Literature
Source: 3D Print Med. 2020 Nov 10;6:34. doi: 10.1186/s41205-020-00087-0 (PMC7653713; doi:10.1186/s41205-020-00087-0)

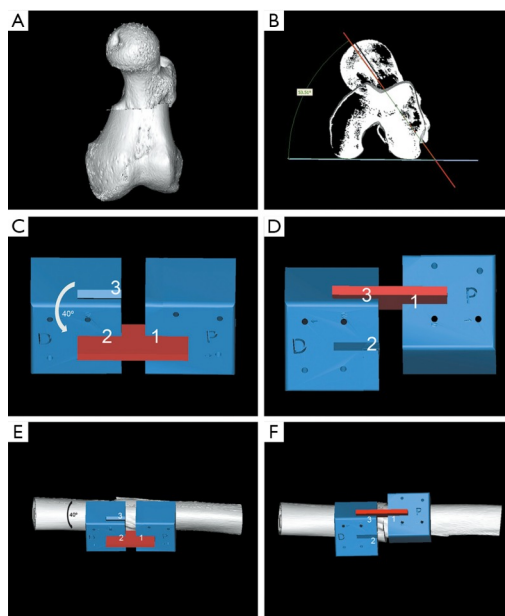

Supplement: Supplementary file 2 — Additional file 2. Image. [file 41205_2020_87_MOESM2_ESM.zip › PSIimage1.pdf]
